# Experimental enhancement of neurphysiological function

**DOI:** 10.3389/fnsys.2014.00189

**Published:** 2014-10-08

**Authors:** Diana Deca, Randal A. Koene

**Affiliations:** ^1^Center for Integrated Protein Science and SyNergy Cluster, Institute of Neuroscience, Technical University MunichMunich, Germany; ^2^Carboncopies.org FoundationSan Francisco, CA, USA

**Keywords:** experimental enhancement, optogenetics, single cell electrical stimulation, whole brain emulation, artificial general intelligence

Enhancing brain function entails controlling neuronal function. There are several methods available for this which led to some relevant experimental data. Deca (2011) Since methods for connectome (Briggman et al., 2011; Prevedel et al., 2014) and circuit functional analysis (Marblestone et al., 2013) are advancing rapidly (Deca, 2012), it makes sense to consider only the most convincing neurophysiological data in the context of enhancement and their future development.

## Stimulation methods: electrical and optical

The Brecht lab (Houweling and Brecht, 2008) has achieved training of a biological neural network in the living animal through a single neuron leading to enhanced learning speed. Microstimulation of the monkey frontal eye fields (FEF) (Goldberg et al., 1986) and training (Ferrera and Lisberger, 1995) can induce eye fixation and use neuronal activity as a predictor for saccadic eye movements (Shadlen and Newsome, 2001). Schiller and Tehovnik mapped the neurophysiological basis of saccadic eye movements (Tehovnik and Lee, 1993) as a basis for a visual prosthetic (Schiller and Tehovnik, 2008).

Optogenetics is by now a stock neuromodulation technique. The Deisseroth lab used it to enhance neuronal direction selectivity through optical stimulation of interneurons (Lee et al., 2012). Increasing inhibition can promote learning. It was also used to modulate the astroglial activation (Perea et al., 2014) for enhancing both excitatory and inhibitory neurotransmission. Neuronal activity can also be inhibited optogenetically (Zhang et al., 2007) using halorhodopsin.

## Neurofeedback

Romo et al. (2000) used microstimulation as a substitute for sensory stimulation and obtained the same results, showing that sensory input can be replaced in a network by its corresponding electrical input. Furthermore, it was shown that rhesus monkeys can control the activity of their own FEF neurons, when experimenters reinforce visual attention (neurofeedback training Schafer and Moore, 2011).

The finding that rats can press a lever in order to get drugs that interfere with their own dopaminergic system (Yokel and Wise, 1976; Wise et al., 1990) also inspired the invention of an electrode for chronic brain self-stimulation.

## Neural prosthetics

The discovery of neural population coding of directional motor control signals (Georgopoulos et al., 1982, 1986), plus the discovery of stable cortical maps for motor control (Ganguly and Carmena, 2009), have enabled control of prosthetic limbs through chronic multi-site neural interfaces in non-human primates (Nicolelis, 2001; Graziano et al., 2002; Nicolelis et al., 2003; Gilja et al., 2012) and human experiments with implantable devices that enable control of a cursor, a wheel chair, a TV remote control, and a prosthetic hand by a single neuron or by an ensemble of neurons (Kennedy and Bakay, 1998; Hochberg et al., 2006; Truccolo et al., 2008; Simeral et al., 2011). There are also efforts to use signals from higher-level cognitive processing to instruct devices (Andersen et al., 2004). The FDA has approved clinical trials for cortical motor control of prosthetic arms using Utah arrays (Maynard et al., 1997).

Work from the Schreiner lab (Atencio et al., 2014) shows that an auditory implant in the thalamus can give better results than cochlear implants.

Also, a short-term memory neuroprosthetic in the rodent hippocampus enhanced performance (Berger et al., 2011). It performed real-time diagnosis and stimulation and enhanced cognitive, mnemonic processes. Furthermore, one can transfer performance-related spiking activity from one donor brain and use this pattern to stimulate another and generate the same behavior through BMBI. Deadwyler et al. (2013), Opris et al. (2001, 2013), Opris and Casanova (2014), Berger and Deadwyler made a neuroprosthetic multi-input multi-output (MIMO) model replicating CA3-to-CA3 coding functions which successfully enhanced monkeys' performance on a decision making task (Dibazar et al., 2013; Hampson et al., 2013) and recovered it under pharmacological disruption (Hampson et al., 2012). They are currently starting trials in volunteer human patients. Guggenmos et al. (2013) invented a prosthetic for restoring motor function. Circuit function was also emulated in the cerebellum (Herreros et al., 2014). Using the neuroprosthetic system, a rat underwent acquisition, retention and extinction of the eye-blink reflex even under anesthesia.

**Table 1:** **Summary of successful neurophysiological enhancements**.

**Enhanced function**	**Method**	**What is modulated**	**Possible developments**
Vision/Stimulus selectivity	Optogenetics	Interneurons	Enhancing other senses and learning by inhibiting the responsible inhibitory circuits
Vision/Stimulus selectivity	Optogenetics	Astrocytes	Speeding up network computation in response to any stimulus by activating the brain's immune response
Learning/Decision making	Single neuron electrical stimulation	Neuronal firing/Behavior	Enhancing a desired behavioral response through electrical stimulation
Oculomotor control	Neurofeedback training	Neuronal firing in the FAF/thalamic	Inducing long-term plasticity and learning through repetitive neurofeedback training
Hearing	Auditory thalamic implant	Thalamic input	Activated auditory cortex at low electrical current levels
Vision/Fixation	Electrical stimulation	Frontal eye fields	Electrically evoked saccadic eye movements
Memory	Neuroprosthetic	Neuronal firing/behavior	Enhanced mnemonic processes through electrical stimulation
Memory	Neuroprosthetic/Emulated firing patterns	Neuronal firing	Induced memory-related processing
Learning	MIMO	Substituted layer 5 neuronal input	Enhanced performance in a primate decision making task
Motor skills	Brain-machine-brain interface (BMBI)	Bridged damaged neural pathways	Promoted LTP, Restored motor function
Learning	Neuroprosthetic	Restored the eye-blink reflex under anesthesia with BMBI	Induced learning in the cerebellum with neuroprosthetic conditioning

## Toward the connectome

The goal of this paper was to present the clearest experimental evidence of neurophysiological enhancement to date, while employing a very conservative definition of enhancement.

The neural mechanisms for the enhancement effects of drugs, deep brain stimulation or transcranial current stimulation are largely unknown. Microstimulation and optogenetics provide means to control specific system components and study their contribution to a particular brain function. Neuroprosthetics, brain implants, MIMO, BMBI, and neurofeedback training do electrophysiological data acquisition, interpretation and reimplementation which, if successful, show a clear direction of causality of the neurophysiological substrate of sensing, learning, memory and decision making. These approaches provide mechanistic explanations together with clear enhancement of brain functions.

In the near future, more mechanistic/causal electrophysiological data showing enhancement in lower animals will enable further exploration of these mechanisms in primate non-human and human subjects. A significant challenge for non-invasive experimental enhancement is getting around the isolating effects of the skull. Lebedev (2014) if this cannot be achieved, then very small invasive implants (Seo et al., 2013) may be an alternative solution.

### Conflict of interest statement

The authors declare that the research was conducted in the absence of any commercial or financial relationships that could be construed as a potential conflict of interest.
